# Trends in Population Dynamics of *Escherichia coli* Sequence Type 131, Calgary, Alberta, Canada, 2006–2016^1^

**DOI:** 10.3201/eid2612.201221

**Published:** 2020-12

**Authors:** Gisele Peirano, Tarah Lynch, Yasufumi Matsumara, Diego Nobrega, Thomas J. Finn, Rebekah DeVinney, Johann D.D. Pitout

**Affiliations:** University of Calgary Cummings School of Medicine, Calgary, Alberta, Canada (G. Peirano, T. Lynch, T.J. Finn, R. De Vinney, J.D.D. Pitout);; Alberta Precision Laboratories, Calgary (G. Peirano, T. Lynch, J.D.D. Pitout);; Kyoto University Graduate School of Medicine, Kyoto, Japan (Y. Matsumara);; University of Calgary, Calgary (D. Nobrega); University of Pretoria, Pretoria, South Africa (J.D.D. Pitout)

**Keywords:** Escherichia coli, bacteria, bloodstream infections, population surveillance, population dynamics, trends, antimicrobial resistance, sequence type 31, clades, incidence rate, Calgary, Alberta, Canada

## Abstract

Global expansion of antimicrobial drug–resistant *Escherichia coli* sequence type (ST) 131 is unrivaled among human bacteria. Understanding trends among ST131 clades will help with designing prevention strategies. We screened *E. coli* from blood samples (n = 1,784) obtained in Calgary, Alberta, Canada, during 2006, 2012, and 2016 by PCR for ST131 and positive samples (n = 344) underwent whole-genome sequencing. The incidence rate per 100,000 residents increased from 4.91 during 2006 to 12.35 during 2012 and 10.12 during 2016. ST131 belonged to clades A (10%), B (9%), and C (81%). Clades C1-nonM27 and B were common during 2006, and C2 containing *bla*_CTX-M-15_, C1-M27 containing *bla*_CTX-M-27_, and A were responsible for the increase of ST131 during 2012 and 2016. C2 was the most antimicrobial drug–resistant subclade and increased exponentially over time. Eradicating ST131, more specifically the C2 subclade, will lead to considerable public health benefits for persons in Calgary.

*Escherichia coli* sequence type (ST) 131 is the quintessential example of a successful, global, antimicrobial-resistant, high-risk clone among human bacteria (*1*,*2*). Currently, ST131 is the most common global extraintestinal pathogenic *E. coli* (ExPEC) clone; up to 30% of all ExPEC, 60%–90% of fluoroquinolone-resistant ExPEC, and 40%–80% of ExPEC with extended-spectrum β-lactamases [ESBLs] belong to ST131 (*3*,*4*). Population genetics indicate that ST131 consists of different clades (*5*): clade A contains serotype O16:H5 and *fimH*41, clade B contains mostly serotype O25b:H4 and *fimH*22, and clade C contains serotype O25b:H4 and *fimH*30. Clade C is divided into 2 subclades: C1/H30R (associated with fluoroquinolone resistance) and C2/H30Rx (associated with fluoroquinolone resistance and *bla*_CTX-M-15_). A novel ST131 C1 subclade, known as C1-M27 with *bla*_CTX-M-27_, was reported in Japan (*6*).

ST131 is the most dominant and most antimicrobial-resistant among *E. coli* causing bloodstream infections in Calgary, Alberta, Canada, infecting mostly the elderly in long-term care centers (*7*). Previous molecular epidemiology studies from the same region showed that ST131 was relatively rare among ESBL-producing and fluoroquinolone-resistant *E. coli* during the early 2000s but showed a major increase toward the end of the 2000s (*8*,*9*). However, limited information is available regarding the changes in population dynamics of ST131 clades over extended periods, especially among nonbiased *E. coli* isolates in large, well-defined, geographic regions.

To address this issue, we conducted a retrospective cohort study that characterized ST131 clades responsible for bloodstream infections in Calgary over an 11-year period (2006–2016). Investigating trends of ST131 clades over long periods by using a population-based surveillance approach will aid in clarifying the evolution of this clone and help with designing superior prevention strategies (*3*,*10*).

## Materials and Methods

### Study Population

We conducted a retrospective cohort study in Calgary by using all *E. coli* human clinical isolates from blood cultures processed by a centralized laboratory system (Alberta Precision Laboratories) during 2006, 2012, and 2016. All blood culture samples from adults and children in inpatient and outpatient settings were included.

### Clinical Data

Clinical information corresponding to source patients at the time of the *E. coli* bloodstream infection was obtained by using Sunrise Clinical Manager (Allscripts Healthcare Solutions, Inc., https://www.allscripts.com). A case-patient with an *E. coli* bloodstream infection was defined as a patient with systemic inflammatory response and documented growth of an *E. coli* isolate in a blood culture. Incident case-patients were defined as Calgary residents with a first isolation of *E. coli* from blood. Repeat *E. coli* from blood were excluded. Bloodstream infections were defined as community acquired, hospital acquired, or healthcare associated (*11*).

### Bacterial Isolates, Identification, and Susceptibility Testing

All *E. coli* isolates from blood were routinely stored at Alberta Precision Laboratories and available for this study. Unique isolates recovered during January 1–December 31, 2006, 2012, and 2016 were obtained from the frozen depository.

Identification was conducted by using matrix-assisted laser desorption/ionization time-of-flight mass spectrometry (Vitek; bioMérieux, https://www.biomerieux.com), and susceptibility testing was conducted the VITEK 2 Instrument (bioMérieux). Susceptibilities were determined for amoxicillin/clavulanic acid, piperacillin/tazobactam, ceftriaxone, meropenem, ertapenem, amikacin, gentamicin, tobramycin, ciprofloxacin, and trimethoprim/sulfamethoxazole. Throughout this study, results were interpreted by using the Clinical Laboratory Standards Institute criteria for broth dilution (*12*). Antimicrobial resistance and virulence scores were determined as described (*13*).

### Molecular Characterization

All *E. coli* isolates (n = 1,786) were initially screened with a PCR specific for ST131 (*14*). Positive isolates (n = 344) underwent whole-genome sequencing, by using procedures previously (*15*,*16*). The Nextera XT DNA Sample Preparation Kit (Illumina, https://www.illumina.com) was used to prepare libraries for sequencing. Samples were multiplexed and sequenced on an Illumina NextSeq500 for 300 cycles (151-bp paired-end). Draft genomes were obtained by using SPAdes version 3.10.1 (*17*). To define the presence of genes and mutations, BLAST (*18*) in combination with following databases or typing schemes were accessed: National Center for Biotechnology Information Bacterial Antimicrobial Resistance Reference Gene Database (https://www.ncbi.nlm.nih.gov/bioproject/PRJNA313047), ResFinder (*19*), PlasmidFinder (*20*), MLST (*21*) virulence finder (*22*), and virulence factor database (*23*). ST131 clades were identified by using an in silico PCR and primers described elsewhere (*14*).

### Statistical Analysis

The Fisher exact test was used to perform pairwise comparisons of factors between clades, *t*-test was used for age comparisons, and p values obtained within individual categories were adjusted for multiple comparisons by using the false discovery rate (*24*). Population data were extracted from census reports from Statistics Canada (https://www.statcan.gc.ca) and used to estimate incidence rates (IRs) on the basis of a Poisson distribution. The Mann-Whitney test was used to compare antimicrobial resistance and virulence scores between clades. The effect of eliminating subclade C2 on nonsusceptibility and IRs was assessed by using Fisher exact and Poisson tests, for which population characteristics were compared with the presence and absence of subclade C2 isolates. The p values were adjusted for multiple comparisons accordingly. All analyses were conducted in R version 3.6.1 (*25*). Statistical significance was set at the 5% level.

### Sequence Data Accession Numbers and Ethics

Sequencing data was deposited in the National Center for Biotechnology Information database (submission no. SUB7225977). This study was approved by the University of Calgary Conjoint Health Research Ethics Board (REB16-2457).

## Results

### *E. coli* Isolates

*E. coli* was the most common bacterium obtained from blood in the Calgary region during 2006 (482 [28.9%] of 1,669 isolates), 2012 (691 [29.7%] of 2,084 isolates), and 2016 (685 [31.1%] of 2,201 isolates). A total of 1,786 unique *E. coli* were screened for ST131: 481 from 2006, 621 from 2012, and 684 from 2016. Overall, 344 (19.2%) of 1,786 *E. coli* isolates were PCR positive for ST131; the prevalence of ST131 increased from 53 (11%) of 481 during 2006 to 150 (24.2%) of 621 during 2012 and 141 (20.6%) of 684 during 2016 (p<0.001 for both comparisons).

Most ST131 isolates belonged to clade C in the following subclades (Table 1): C0 (n = 5, 2%), C1-nonM27 (n = 121, 35%), C1-M27 (n = 13, 4%), and C2 (n = 139, 40%). The remainder of ST131 isolates belonged to clades A (n = 34 [10%)] and B (n = 32 [9%]).

**Table 1:** Patient characteristics associated with *Escherichia coli* sequence type 131 clades, Calgary, Alberta, Canada, 2006–2016*

Characteristic	Clade						
	A, n = 34	B, n = 32	C0, n = 5	C1-nonM27, n = 121	C1-M27, n = 13	C2, n = 139	All, n = 344
Year							
2006	0^a^	14 (44)^b,c^	5 (100)^b^	24 (20)^c^	1 (8)^a,c^	9 (7)^a^	53 (15)
2012	18 (53)	9 (28)	0	56 (46)	4 (31)	63 (45)	150 (44)
2016	16 (47)	9 (28)	0	41 (34)	8 (34)	67 (48)	141 (41)
Sex							
M	14 (41)	10 (31)	3 (60)	59 (49)	9 (69)	76 (55)	171 (50)
F	20 (59)	22 (69)	2 (40)	62 (57)	4 (31)	63 (45)	173 (50)
Mean age, y (range)	52.9 (1–87)	66 (8–103)	72 (57–79)	67.8 (1–95)	73.1 (45–92)	67.8 (9–94)	
Clinical infection							
Primary sepsis	13 (38)^a^	9 (28)	4 (80)^a^	15 (12)^b^	6 (46)	22 (16)	69 (20)
Urinary tract	15 (44)	14 (44)	1 (20)	66 (55)	6 (46)	84 (84)	186 (54)
Acute biliary	2 (6)	1 (3)	0	17 (1)	1 (8%)	10 (7)	31 (9)
Intraabdominal	3 (9)	4 (1)	0	7 (6)	0	9 (7)	23 (7)
Pneumonia	1 (3)	4 (13)	0	16 (13)	0	14 (10)	35 (10)
Origin of infection							
Community-acquired	16 (47)	23 (72)^a^	4 (8)	31 (26)^b^	4 (31)	40 (29)^b^	118 (34)
Healthcare-associated	11 (32)	7 (22)^a^	0	70 (58)^b^	9 (69)	69 (50)	166 (48)
Hospital-acquired	7 (21)	2 (6)	1 (20)	20 (16)	0	30 (21)	60 (18)
Not susceptible							
AMC	4 (12)^a^	5 (16)^a^	0^a^	34 (28)^a^	0^a^	85 (61)^b^	128 (37)
TZP	0	0	0	7 (6)	0	15 (11)	22 (6)
CRO	4 (12)^a,b^	1 (3)^b^	0^a,b,d^	40 (33)^a,d^	7 (54)^c,d^	94 (68)^c^	146 (42)
ERT/MEM	0	0	0	0	0	1 (1)	1 (0.3)
CIP	5 (1)^a^	0^a^	1 (20)^a^	121 (100)^b^	13 (100)^b^	139 (100)^b^	279 (81)
SXT	22 (65)	13 (41)	3 (60)	70 (58)	6 (46)	67 (48)	181 (53)
GEN	11 (32)	8 (25)	0	59 (49)^a^	0^b^	62 (45)^a^	140 (41)
TOB	10 (29)^a,b^	7 (22)^a,b^	0^a,b^	53 (44)^a^	0^b^	86 (62)^c^	156 (45)
AMK	0	0	0	0	0	1 (1)	1 (0.3)
Resistance score, median (range)†	1^a,b^ (0–4)	1^b^ (0–4)	1^a,b^ (0–1)	3^c^ (1–5)	2^a,c^ (1–3)	4^d^ (1–5)	
Serotype							
O16:H5	34 (100)^a^	0^b^	0^b^	0^b^	0^b^	0^b^	34 (10)
O25:H4	0^a^	31 (97)^b^	5 (100)^b^	121 (100)^b^	13 (100)^b^	139 (100)^b^	309 (90)
O2H4	0	1 (3)	0	0	0	0	1 (0.3)
*fimH*							
41	33 (97)^a^	0^b^	0^b^	0^b^	0^b^	0^b^	33 (10)
89	1 (3)	0	0	0	0	0	1 (0.3)
22	0^a^	15 (47)^b^	0	0^a^	0^a^	0^a^	15 (4)
27	0^a^	12 (38)^b^	0	0^a^	0^a^	0^a^	12 (3)
324	0	1 (3)	0	0	0	0	1 (0.)
30	0^a^	4 (13)^a^	5 (100)^b^	121 (100)^b^	13 (100)^b^	139 (100)^b^	282 (82)
Plasmid type							
Col (B551)	0	0	0	17 (14)	3 (23)	21 (15)	41 (12)
Col (MG828)	0	0	0	3 (2)	1 (8)	13 (9)	17 (8)
Col156	0^a^	8 (25)^b^	0	2 (2)^a^	1 (8%)	22 (16)^b^	33 (10)
Col8282	0	0	0	0	0	1 (1)	1 (0.3)
FIA	8 (32)^a,b^	1 (3)^a^	4 (80)^b,c^	110 (91)^c^	13 (100)^c^	125 (90)^c^	261 (76)
FIB	33 (97)^a^	28 (88)	4 (80)	102 (84)^a^	12 (92)	98 (71)^b^	277 (81)
FIC	1 (3)	7 (22)	0	6 (5)	0	35 (25)	49 (14)
FII	26 (76)	10 (31)	3 (60)	23 (19)	1 (3)	83 (60)	146 (42)
IncI1	3 (9)	2 (6)	1 (20)	16 (13)	1 (3)	8 (6)	31 (9)
IncN	1 (3)	1 (3)	1 (20)	6 (5)	1 (3)	7 (5)	17 (5)
IncX1	0	2 (6)	0	2 (2)	0	1 (1)	5 (1)
IncX4	1 (3)	1 (3)	0	6 (5)	2 (15)	9 (6)	19 (6)
IncY	0	0	0	0	0	7 (5)	7 (2)
IncF replicons							
FII_1	6 (18)^a^	8 (25)^a^	0^a^	92 (76)^b^	12 (92)^b^	16 (12)^a^	134 (39)
FII_2	5 (15)^a^	1 (3)^a,c^	0^a,c^	105 (87)^b^	13 (100)^b^	2 (1)^c^	126 (37)
FIB_20	6 (1)^a,c^	1 (3)^a,b^	3 (60)^c,d^	97 (80)^d^	11 (85)^d^	2 (1)^b^	120 (35)
FII_2	1 (3)^a^	8 (25)^b^	1 (20)	19 (16)^a,b^	1 (8)^a,b^	63 (45)^c^	93 (27)
FIA_1	3 (9)^a^	0^a^	4 (80)^b^	2 (2)^a^	0^a^	69 (50)^b^	78 (23)
Grouped plasmids							
Group 1	5 (15)^a^	1 (3)^a,b^	0^a,b^	80 (66)^c^	11 (85)^c^	0^b^	98 (28)
Group 2	0^a^	0^a^	1 (20)	0^a^	0^a^	56 (40)^b^	57 (17)

*Values are no. (%) unless indicated otherwise. Not susceptible include intermediate or resistant rates. Rates followed by different superscript letters indicate significant differences between clades at the 5% level (adjusted for multiple comparisons). Group 1 plasmids: combination of FIA_1, FII_2, and FIB_20. Group 2 plasmids: combination of FII_2 and FIA_1. AMC, amoxicillin/clavulanate; AMK, amikacin; CIP, ciprofloxacin; CRO, ceftriaxone; ERT, ertapenem; GEN, gentamicin; MEM, meropenem; SXT, trimethoprim/sulfamethoxazole; TOB, tobramycin; TZP, piperacillin/tazobactam. †The resistance score was the number of antimicrobial drug classes to which resistance was detected.

### Incidence Rates and Population Dynamics of ST131 Clades

The IR per 100,000 residents with ST131 bloodstream infections in Calgary increased from 4.91 during 2006 to 12.35 during 2012 and 10.12 during 2016 (p<0.001 for both comparisons). Overall, the population structure of ST131 was dominated by the C clade. However, the IRs per 100,000 residents and proportions among the different subclades showed a major change over time (Table 2; Figure). The C0 subclade represented 9.4% of the ST131 population during 2006, with an estimated IR of 0.46 cases per 100,000 residents. However, the C0 subclade was not detected during 2012 and 2016 (p = 0.001 for both comparisons).

**Table 2:** Incidence rates/100,000 residents for *Escherichia coli* sequence type 131 clades, Calgary, Alberta, Canada, 2006, 2012, and 2016*

Clade	IR (95% CI)		
	2006	2012	2016
A	0.00^a^ (0.00–0.34)	1.48^b^ (0.88–2.34)	1.15^b^ (0.66–1.87)
B	1.30 (0.71–2.18)	0.74 (0.34–1.41	0.65 (0.30–1.23)
C0	0.46^a^ (0.15–1.08)	0.00^b^ (0.00–0.30)	0.00^b^ (0.00–0.26)
C1-nonM27	2.22^a^ (1.55–3.60)	4.61^b^ (3.48–5.99)	2.94^a^ (2.11–3.99)
C1-M27	0.09^a^ (0.00–0.56)	0.33 (0.09–0.84)	0.57^b^ (0.25–1.13)
C2	0.83^a^ (0.41–1.72)	5.19^b^ (3.98–6.63)	4.81^b^ (3.73–6.11)
Total	4.91^a^ (3.68–6.42)	12.35^b^(10.45–14.49)	10.12^b^ (8.52–11.94)

*Rates followed by different superscript letters indicate significant differences between years at the 5% level. IR; incidence rate.

**Figure:**
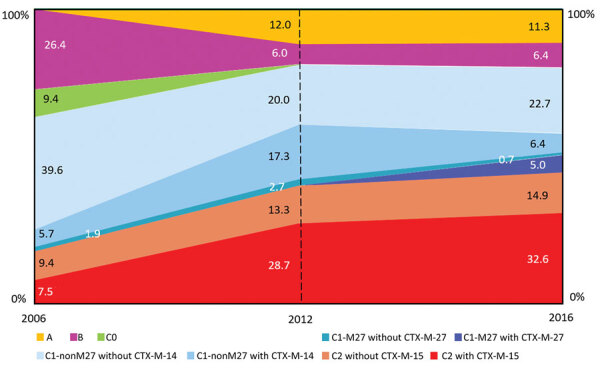
Proportions of *Escherichia coli* sequence type 131 clades, Calgary, Alberta, Canada, 2006–2016.

The C1-nonM27 subclade dominated the population structure of ST131 during 2006 (comprising of 46% of the total population, with an IR of 2.22/100,000 residents). Despite an increased IR during 2012 and 2016 (when compared with that for 2006), the frequency of C1-nonM27 isolates decreased to 37.3% in 2012 and 29% in 2016 (2006 vs. 2016; p = 0.04) (Figure). The C1-M27 subclade increased from 1.9% during 2006 to 5.7% during 2016. There was an association between C1-M27, the presence of *bla*_CTX-M-27_, and year of isolation (5 C1-M27 isolates from 2006 and 2012 were negative for *bla*_CTX-M-27_, and 7/8 isolates obtained during 2016 were positive for *bla*_CTX-M-27_) (p = 0.004) (Figure).

The prevalence of C2 subclade increased substantially from 17% of the total ST131 population during 2006 to 42% during 2012 and 47% during 2016 (p<0.001 for both comparisons) (Figure). The IR per 100,000 residents of the C2 clade increased from 0.83 during 2006 to 5.19 during 2012 and 4.81 during 2016 (p<0.001 for both comparisons) (Table 2). The increase in subclade C2 correlated with the presence of CTX-M-15 (4 [44%] of 9 of C2 isolates from 2006 were positive for *bla*_CTX-M-15_ compared with 89 [68%] of 130 isolates obtained during 2012 and 2016) (Figure).

Clade A was absent among ST131 during 2006 and then increased to 12% of the ST131 population during 2012 and 11.3% of the ST131 population during 2016 (p<0.01 for both comparisons) (Figure). The IR of clade A increased from 0 to 1.48/100,00 residents during 2012 and to 1.15/100,000, residents during 2016 (p<0.001 for both comparisons) (Table 2). B was the second most common clade during 2006 (26.4% of the total ST131 population), but decreased to 6% of the ST131 population during 2012 and to 6.4% of the ST131 population during 2016 (p<0.001 for both comparisons) (Figure). The IR of clade B decreased from 1.30/100,000 residents to 0.74/100,000 residents during 2012 and to 0.65/100,000 residents during 2016 (Table 2).

### Clinical Characteristics

*E. coli* ST131 bloodstream infections were evenly distributed between male patients (n = 171, 49.7%) and female patients (n = 173, 50.3%) (Table 1). Just under half (48%) of *E. coli* ST131 bloodstream infections were healthcare-associated, followed by community-acquired (34%) and hospital-acquired (18%) (Table 1). Clades A and B were associated with community-acquired infections, and patients infected with clade C were more likely to be healthcare associated. Patients infected with clade A tended to be younger (Table 1). More than half (n = 186, 54%) of patients had upper urinary tract infections, followed by bloodstream infections with an unknown source (n = 69, 20%), pneumonia (n = 35, 10%), acute biliary tract infections (n = 31, 9%), and intraabdominal infections (n = 23, 7%) (Table 1).

### Serotypes, *fim*H Types, and Antimicrobial Susceptibilities

Clade A contained O16:H5, *fim*H41, and *fim*H89. Clade B contained O25:H4, O2:H4, *fim*H22, *fim*H27, *fim*H324, and *fim*H30. Clade C contained O25:H4 and *fim*H30 (Table 1).

Overall, high (>25%), intermediate, or resistant (not susceptible) rates were observed for ceftriaxone, ciprofloxacin, trimethoprim/sulfamethoxazole, gentamicin, and tobramycin. Low rates (<5%) were observed for amikacin, ertapenem, and meropenem. C2 was the most antimicrobial-resistant subclade, followed by C1-nonM27 and C1-M27 (Table 1). Clades B and C0 were the most susceptible clades, and clade A showed high nonsusceptible rates for trimethoprim/sulfamethoxazole, gentamicin, and tobramycin (Table 1).

### Removal of Subclade C2

Eliminating subclade C2 would have decreased the incidence rate of ST131 bloodstream infections from 12.35/100,000 residents to 7.16/100,000, residents during 2012 and from 10.12/100,00 residents to 5.31/100,000 residents during 2016 (p<0.001 both comparisons). In addition, eliminating subclade C2 would have resulted in a significant reduction of not susceptible rates for amoxicillin/clavulanic acid, ciprofloxacin, ceftriaxone, and tobramycin for ST131 causing bloodstream infections in Calgary (2006, 2012, and 2016) (p<0.05 for all comparisons).

### Quinolone Resistance–Determining Regions and Antimicrobial Resistance Determinants

The combination of mutations in gyrase A genes (*gyrA* S83L and *gyrA* D87N) and DNA topoisomerase IV genes (*parC* S80I, *parC* E84V, and *parE* I529L) in the quinolone resistance-determining regions were present in all C1 and C2 isolates (Table 3). Nearly all (97%) ST131 isolates contained the *parE* I529L mutation. Most (85%) clade A isolates had the *gyrA* S83L mutation; for 5 isolates, this mutation was combined with *gyrA* D87N and *parC* S80I, and 1 isolate had the *gyrA* S83L, *gyrA* D87N, *parC* S80I, and *parC* E84V combination. Mutations in *gyrA* and *parC* were rare in clade B; 2/32 isolates had the *gyrA* S83L and *parC* S80I mutation combination (Table 3). One subclade C0 isolate had only the *gyrA* S83L mutation, and another C0 isolate had the *gyrA* S83L, *gyrA* D87N, *parC* S80I, and *parC* E84V combination.

**Table 3:** Factors associated with *Escherichia coli* sequence type 131 clades, Calgary, Alberta, Canada, 2006, 2012, and 2016*

Factor	Clade						
	A, n = 34	B, n = 32	C0, n = 5	C1-non-M27, n = 121	C1-M27, n = 13	C2, n = 139	All, n = 344
QRDR mutation							
* gyrA* S83L	29 (85)^a,b^	2 (6)^c^	2 (40)^b,c^	121 (100)^d^	13 (100)^a,d^	139 (100)^d^	306 (89)
* gyrA* D87N	5 (15)^a^	0^a^	1 (20)^a^	121 (100)^b^	13 (100)^b^	139 (100)^b^	279 (8)
* parC* S80I	5 (15)^a^	2 (6)^a^	1 (20)^a^	121 (100)^b^	13 (100)^b^	139 (100)^b^	281 (82)
* parC* E84V	1 (3)^a^	0^a^	1 (20)^a^	121 (100)^b^	13 (100)^b^	139 (100)^b^	275 (80)
* parE* I529L	30 (88)^a^	27 (84)^a^	5 (100)	121 (100)^b^	13 (100)	139 (100)^b^	335 (97)
β-lactamase							
CTX-M-15	2 (6)^a^	1 (3)^a^	0^a^	1 (1)^a^	0^a^	93 (67)^b^	97 (28)
CTX-M-14	0^a^	0^a^	0	38 (31)^b^	0	1 (1)^a^	39 (11)
CTX-M-27	1 (3)^a^	0^a^	0	0^a^	7 (54)^b^	0^a^	8 (2)
CTX-M-55	2 (6)	0	0	0	0	0	2 (0.6)
CTX-M-198	0	0	0	1 (1)	0	0	1 (0.3)
NDM-5	0	0	0	0	0	1 (1)	1 (0.3)
OXA-1	0^a^	0^a^	0^a^	1 (1)^a^	0^a^	84 (6)^b^	85 (25)
OXA-9	0	0	0	0	0	1 (1)	1 (0.3)
SHV-12	0	0	0	0	0	2 (1)	2 (0.6)
TEM-1	29 (85)^a,b^	20 (63)^a^	4 (80)^a,b^	103 (85)^b^	1 (8)^c^	26 (19)^c^	183 (53)
TEM other	0	0	0	2 (2)	0	2 (1)	4 (1)
CMY-2	0	2 (6%)	0	1 (1)	0	0	3 (0.9)
Aminoglycoside-modifying enzyme							
* Aac(3)-IIa*	0^a^	0^a^	0	2 (1)^a^	0^a^	57 (41)^b^	59 (17)
* Aac(3))-IId*	11 (32)^a,b^	15 (47)^a^	0	63 (52)^a^	0^b,c^	4 (3)^c^	93 (27)
* aac(6')-Ib-cr*	0^a^	0^a^	0^a^	2 (2)^a^	0^a^	84 (60)^b^	86 (25)
* aadA1*	0	2 6)	0	0	0	4 (3)	6 (2)
* aadA16*	0	0	0	1 (1)	0	1 (1)	2 (0.6)
* aadA2*	0^a^	14 (44)^b^	0	0^a^	0^a^	2 (1)^a^	16 ()
* aadA5*	23 (68)^a^	1 (3)^b^	3 (60)^a,c^	70 (58)^a^	7 (54)^a,c^	57 (41)^c^	161 (47)
* ant(2′′)-Ia*	0	0	0	0	0	5 (4)	5 (1)
* aph(3′)-Ia*	1 (3)	2	0	1 (11)	0	0	4 (1)
* aph(3′′)-Ib*	20 (59)^a^	3 (9)^b^	0	69 (57)^a^	5 (38)	26 (19)^b^	123 (36)
* aph(3′)-IIa*	0	1 (3)	0	1 (1)	0	0	2 (0.6)
* Aph(6)-Ic*	0	1 (3)	0	1 (1)	0	0	2 (0.6)
* Aph(6)-Id*	20 (59)^a^	3 (9)^b^	0	68 (56)^a^	5 (38)	25 (18)^b^	121 (35)
Other							
* qnrB*	0	0	0	0	0	2 (1)	2 (0.6)
* ARR-3*	0	0	0	1 (1)	0	1 (1)	2 (0.6)
* dfrA1*	0	1 (3)	0	0	0	0	1 (0.3)
* dfrA12*	0^a^	13 (41)^b^	0	0^a^	0^a^	2 (1)^a^	15 (4)
* dfrA14*	1 (3)	0	0	0	0	8	12 (3)
* dfrA17*	22 (65)^a^	1 (3)^b^	3 (60)^a^	70 (58)^a^	7 (54)^a^	59 (42)^a^	162 (47)
* dfrA27*	0	0	0	1 (1)	0	1 (1)	2 (0.6)
* dfrA5*	0	1 (3)	0	0	0	0	1 (0.3)
* sul1*	22 (65)	15 (47)	3 (60)	69 (57)	6 (46)	63 (45)	178 (52)
* sul2*	20 (59)^a^	3 (9)^b^	0	70 (58)^a^	5 (38)	28 (20)^b^	126 (37)
* sul3*	0	1 (3)	0	0	0	0	1 (0.3)
* tetA*	19 (56)^a^	5 (16)^b^	0	61 (50)^a^	6 (4)	77 (55)^a^	168 (49)
* tetB*	2 (6)	3 (9)	0	2 (2)	0	3 (2)	10 (3)

*Values are no. (%). Rates followed by different superscript letters indicate significant differences between clades at the 5% level (adjusted for multiple comparisons).

CTX-M β-lactamases were detected among 148 (43%) isolates; most were CTX-M-15, followed by CTX-M-14, CTX-M-27, CTX-M-55, and CTX-M-198 (Table 3). CTX-M types were associated with different subclades (e.g., *bla*_CTX-M-14_ with C1-nonM27, *bla*_CTX-M-15_ with C2, *bla*_CTX-M-27_ with C1-M27, and *bla*_CTX-M-55_ with A). TEM-1 was common in most clades, with the exception of C2 and C1-M27. Three ST131 isolates were positive for *bla*_CMY-2_, and 1 C2 isolate was positive for *bla*_NDM-5_.

Certain aminoglycoside-modifying enzymes were common among ST131: *aac(**3**)-IId, aac(6¢)-Ib-cr*, *aadA5, aph(3¢¢)-Ib*, and *aph (**6**)-Id* (Table 3). Some associations between presences of aminoglycoside-modifying enzymes with certain subclades were noted: *aac (**3**)-IId* were present mainly in clades A, B, and C1-nonM27; *aac(6')-Ib-cr* in subclade C2; *aadA2* in clade B, and *aadA5* in clades A, C0, and C1. The combination of *aph(3¢¢)-Ib* and *aph (**6**)-Id* was more common in clades A and C1-nonM27 (Table 3). With regard to the presence of other antimicrobial resistance determinants, *qnr* was rare, and *dfrA17*, *sul1*, *sul2*, and *tetA* were common among most of ST131 clades (Table 3).

### Plasmids and Replicon Types

Overall, IncF plasmid types (e.g., combinations of FIA, FIB, FIC, and FII) were common among all ST131 clades. Col-like plasmids and other plasmid families (IncI1, IncN, IncX1, IncX4, and IncY) were widely distributed across all clades but less common than IncF types (Table 1). 

Using IncF plasmid replicons (FII_1, FIA_2, FIB_20, FII_2, and FIA_1) and a plasmid classification system published recently (*26*), we found that group 1 plasmids (combination of FII_1, FIA_2, and FIB_20) were in clades A, B, C1-nonM27, and C1-M27, and group 2 plasmids (combination of FII_2 and FIA_1) were in clades C0 and C2 (Table 1). Group 1 plasmids were common among C1 clades, and group 2 plasmids were common among C2 isolates.

### Virulence-Associated Factors

The presence of 37 putative virulence factors were assessed for different clades (Table 4). The following factors were present among most isolates: *papAIX*, *iha*, *fimH*, *sat*, *fyuA*, *usp*, *iss*, and *malX*. Some virulence factors were associated with certain clades: *pap*BCFJK, *iha*, *hlyA*, and *cnf1* with subclade C2; *afaABCD*, *draABCDP* *vat*, and *traT* with clade A; *afaABCD*, *draABCDP*, *kpsMII*, and *ibeABC* with clade B; and *kpsMT*III with subclades C0 and C1. No major differences in virulence scores were observed for the different clades.

**Table 4:** Virulence factors associated with *Escherichia coli* sequence type 131 clades, Calgary, Alberta, Canada, 2006, 2012, and 2016*

Factor	Clade						
	A, n = 34	B, n = 32	C0, n = 5	C1-nonM27, n = 121	C1-M27, n = 13	C2, n = 139	All, n = 344
Adhesion gene							
* papA*	34 (100)	28 (88)	5 (100)	121 (100)	13 (100)	139 (100)	340 (99)
* papB*	1 (3)^a^	11 (34)	0	35 (29)^a^	1 (8)	90 (65)^b^	138 (40)
* papC*	1 (3)^a^	13 (41)	0	31 (26)^a^	2 (16)	89 (64)^b^	141 (41)
* papD*	1 (3)^a^	13 (41)	0	36 (30)^a^	1 (8)	89 (64)^b^	140 (41)
* papE*	1 (3)	11 (34)	0	0	0	9 (6)	21 (6)
* papF*	2 (6)^a^	12 (38)	0	34 (28)^a^	1 (8)	90 (65)^b^	139 (40)
* papG*	1 (3)	14 (44)	0	34 (28)	1 (8)	91 (6)	141 (41)
* papH*	1 (3)	9 (28)	0	33 (27)	0	41 (29)	84 (24)
* papI*	32 (94)	24 (75)^a^	5 (100)	116 (96)	12 (92)	137 (99)^b^	326 (95)
* papJ*	1 (3)^a^	11 (34)	0	34 (28)^a^	1 (8)	90 (65)^b^	137 (40)
* papK*	1 (3)^a^	11 (34)	0	34 (28)^a^	1 (8)	90 (65)^b^	137 (40)
* papX*	32 (94)	21 (66)^a^	5 (100)	100 (83)	10 (77)	134 (96)^b^	302 (88)
* Iha*	33 (97)	16 (50)^a^	5 (100)	111 (92)	13 (100)	137 (99)^b^	314 (91)
* fimH*	34 (100)	32 (100)	5 (100)	121 (100)	13 (100)	139 (100)	344 (100)
* Tsh*	0	2 (6)	0	0	0	0	2 (0.6)
* Hra*	1 (1)	0	1 (25)	1 (1)	0	8 (6)	11 (3)
* afaABCD*	18 (53)^a^	18 (56)^a^	1 (25)	4 (3)^b^	0	28 (20)^b^	69 (20)
* draABCDP*	18 (53)^a^	18 (56)^a^	1 (25)	4 (3)^b^	0	28 (20)^b^	69 (20)
Toxin gene							
* hlyA*	1 (1)	11 (34)	0	27 (22)	0	50 (36)	89 (26)
* Sat*	30 (88)	21 (66)^a^	5 (100)	112 (93)	13 (100)	136 (98)^b^	317 (92)
* Vat*	34 (100)^a^	7 (22)^b^	0	1 (1)^b^	0^b^	0^b^	42 (1)
* astA*	1 (3)	1 (3)	0	0	0	6 (4)	8 (2)
* cnf1*	1 (3)	9 (28)	0	29 (24)	0	52 (37)	91 (26)
Siderophore gene							
* iroN*	0	9 (28)	0	1 (1)	0	9 (6)	19 (6)
* fyuA*	34 (100)	32 (100)	5 (100)	121 (100)	13 (100)	139 (100)	344 (100)
* ireA*	0	0	0	0	0	11 (8)	11 (3)
* iutA*	29 (85)	27 (84)	5 (100)	112 (93)	12 (92)	137 (99)	322 (94)
Capsular antigen gene							
*kpsM* II	0	14 (44)	0	0	0	2 (1)	16 (5)
*kpsMT* III	0^a^	13 (41)	5 (100)	71 (59)^a,b^	13 (100)^b^	37 (26)^c^	139 (40)
Miscellaneous gene							
* Usp*	33 (97)	32 (100)	5 (100)	121 (100)	12 (92)	137 (99)	340 (99)
* traT*	28 (82)^a^	13 (41)^b^	0	20 (17)^b^	0^b^	28 (20)^b^	89 (26)
* ompT*	0	4 (13)	0	2 (2)	0	1 (1)	7 (2)
* Iss*	9 (26)^a^	31 (97)	5 (100)	118 (98)^b^	13 (100)	139 (100)^b^	315 (92)
* malX*	34 (100)	32 (100)	5 (100)	118 (98)	13 (100)	138 (99)	340 (99)
* cdtB*	0	2 (6)	0	0	0	0	2 (0.6)
* cvaC*	0	4 (13)	0	0	9 (69%)	10 (7%)	14 (6)
* ibeABC*	0	14 (44)^a^	0	0	0	0^b^	14 (6)
Virulence score, median (range)†	11 (7–15)	10 (6–14)	11 (9–16)	10 (6–15)	10 (9–14)	11 (7–15)	

*Values are no. (%) unless indicated otherwise. Rates followed by different superscript letters indicate significant differences between clades at the 5% level (adjusted for multiple comparisons). *afa,* afimbrial adhesin; *astA*, enteroaggregative *E. coli* toxin; *cdtB,* cytolethal distending toxin B; *cnf1*, cytotoxic necrotizing factor; *cvaC*, factor facilitating colonization; *dra*, Dr binding adhesins; *fimH*, type-1 fimbriae; *fyuA*, yersiniabactin (siderophore) receptor; *hlyA*, α-hemolysin; *hra*, heat-resistant agglutinin; *ibeABC,* invasion of brain endothelium; *iha*, iron-regulated adhesin; *ireA*, iron-regulated element (catecholate siderophore); *iroN*, salmochelin (siderophore) receptor; *iss*, increased serum survival; *iutA*, aerobactin (siderophore) receptor; *kpsM* II, group II capsule variants synthesis; *kpsM* III, group III capsule variants synthesis; *malX*, pathogenicity island marker; *ompT*, outer membrane protein T; *papA*, P fimbriae; *papBCDEFGHIJKX*, genes of P fimbriae operon; *sat*, secreted autotransporter toxin; *traT*, complement inhibition protein; *tsh*, temperature-sensitive hemagglutinin; *usp*, uropathogenic-specific protein; *vat*, vacuolating autotransporter. toxin.  †The virulence gene score was the number of virulence operons detected.

## Discussion

The abrupt global expansion of ST131 during the 2000s is unrivaled among human bacteria and is a real-world model for the evolution of antimicrobial-resistant high-risk clones (*10*). This study describes the clinical features, incidence rates, genomic characteristics, and changes in population structure of ST131 clades causing bloodstream infections in a large centralized region of Canada over an 11-year period (2006–2016). The incidence rates and prevalence of ST131 increased over the time period, mostly caused by an influx of subclades C2 with *bla*_CTX-M-15_ and C1-M27 with *bla*_CTX-M-27_. Such results reinforce the possible role of CTX-M enzymes in the evolutionary success of ST131 (*10*). The presence of *bla*_CTX-M-14_ among C1-nonM27 isolates did not provide a beneficial advantage to this subclade. This finding is probably caused by clonal interference among 2 clones that have acquired different beneficial mutations competing in the same environment (*27*).

The population structure of ST131 in the Calgary region was dominated by clade C, which is similar to results from a previous large global study (*28*). The C clade originated from clade B during the mid to late 1980s by acquisition of several prophages, genomic islands, the *fimH30* allele, and mutations within *gyrA* and *parC* that likely transpired in North America (*29*,*30*). The C clade in this study was mostly responsible for healthcare-associated urinary tract infections. C2 was the most common and most antimicrobial-resistant subclade in this collection and was associated with group 2 plasmids, *bla*_CTX-M-15_ and *aac(6')-Ib-cr*, as well as the virulence factors *iha*, *hlyA*, and *cnf1*. This subclade became prominent during 2012 and 2016 and showed the highest IRs among all subclades during this period. The increase of C2 correlated with the presence of CTX-M-15. Elimination of the C2 subclade through vaccination or phage-therapy programs (*31*), will lead to major decreases in incidence and antimicrobial-resistant rates among ST131 causing bloodstream infections in Calgary.

The C1-nonM27 subclade was the most common subclade during 2006 and associated with group 1 plasmids, *bla*_CTX-M-14_, and *aac (**3**)-IId*. Overall, the C1-M27 subclade was rare (especially during 2006 and 2012) but increased substantially during 2016, which correlated with the presence of *bla*_CTX-M-27_. The C1-M27 subclade has previously been responsible for increases in ESBL-producing *E. coli* from Japan and was also present among ST131 obtained from Thailand, Australia, Canada, and the United States (*6*). The ST131 C1-M27 subclade is currently emerging in Germany (*32*) and France (*33*) and is responsible for 27% of 144 clinical ST131 obtained from different sites in Europe (*34*).

Clade A is likely the ancestral lineage of ST131 and probably originated in Southeast Asia during the mid to late 1880s (*30*). Clade A isolates are generally sensitive to antimicrobial drugs and appear to occupy distinct ecologic niches, such as waste water (*35*). Results from this study show that clade A isolates have high not susceptible rates for trimethoprim/sulfamethoxazole, gentamicin, and tobramycin and were associated with community-associated and healthcare-associated urinary tract infections in younger patients. The virulence factors *afaABCD*, *draABCDP*, *vat*, and *traT* were common in clade A. Also, clade A was absent among ST131 from 2006 but became the third most common clade during 2012 and 2016, replacing clades B and C0 during these periods.

Clade B emerged from clade A in the early 1900s and most likely occurred in North America (*10*,*30*). Members of clade B are antimicrobial susceptible, and several intermediate subclades have been identified (*29*). Our study showed that clade B isolates were the second most common clade during 2006 but decreased substantially during 2016. This clade was the most antimicrobial sensitive ST131 clade in Calgary and was associated with community-acquired urinary tract infections and virulence factors *afaABCD*, *draABCDP*, *kpsMII*, and *ibeABC*.

Previous data have shown that *gyrA S83L* mutations occurred first among fluoroquinolone-resistant *E. coli* and is a major initial step for establishing relative fitness among antimicrobial-resistant isolates (*36*). Our study showed that *gyrA* mutations were rare among clade B isolates, but *parE* I529L mutations were common. This finding suggests that *parE* I529L mutations are the first to occur among fluoroquinolone-resistant ST131. The order in which these mutations arise might play a major role in establishing fitness in ST131 (*37*).

Our study had some limitations. Only patients in Calgary who had positive blood cultures for *E. coli* were included, which excluded those with *E. coli* bloodstream infections from whom no blood samples were submitted for culture. Therefore, incidence rates should be considered as conservative estimates of ST131 bloodstream infections in Calgary, especially for patients infected with clades A and B, who tended to be younger (i.e., clade A infections) and from the community (i.e., clade B infections). Such patients were less likely to have had blood cultures taken than patients who are older or who had previous contact with the healthcare system.

The novel approach for our study used population-based surveillance to describe the incidence rates, specific characteristics, and trends among ST131 clades over an 11-year period in a well-defined human population. We showed major differences in IRs, frequencies, resistance patterns, antimicrobial resistance determinants, grouped plasmid types, virulence factors, and trends over time for different clades. We provided insights into the evolution of ST131 clades in a large well-defined region of Canada. The population structure of ST131 in large geographic healthcare regions is dynamic and has continuous interplay between different subclades.

A previous study showed that eliminating ST131 would substantially decrease the overall IR and antimicrobial-resistant burden within *E. coli* causing bloodstream infections in the Calgary region (*7*). This study identified ST131 subclade C2 as the predominant and most antimicrobial-resistant subclade in Calgary, which is increasing exponentially over time. Eradicating ST131, more specifically the C2 subclade, will lead to considerable public health benefits for persons in Calgary.
